# Identification of a Two-lncRNA Signature with Prognostic and Diagnostic Value for Hepatocellular Carcinoma

**DOI:** 10.1155/2022/2687455

**Published:** 2022-07-21

**Authors:** Huiying Liu, Jianjun Zhu, Lingling Guo, Hongjuan Zhang, Shuxiong Liu, Xiaoxia Kou

**Affiliations:** ^1^Department of Oncology Biotherapy, Eastern Hepatobiliary Surgical Hospital, Shanghai 200433, China; ^2^Department of Medical Oncology, The Sixth People's Hospital of Yancheng, Yancheng 224002, China; ^3^Department of Comprehensive Medicine, Eastern Hepatobiliary Surgical Hospital, Shanghai 200433, China

## Abstract

**Background:**

Accumulating evidence has revealed the important role of long noncoding RNAs (lncRNA) in tumorigenesis and progression of hepatocellular carcinoma (HCC). This study aimed to identify potential lncRNAs that can serve as diagnostic and prognostic signatures for HCC.

**Methods:**

Expression profiling analysis was performed to identify differentially expressed lncRNAs (DElncRNA) between HCC and matched normal samples by integrating two independent microarray datasets. Functional Gene Ontology (GO) terms and Kyoto Encyclopedia of Genes and Genomes (KEGG) pathways were explored by Gene Set Variation Analysis. The prognostic and diagnostic models were developed based on two DElncRNAs. Real-time PCR was used to quantify the relative expressions of candidate lncRNAs.

**Results:**

Two robust DElncRNAs were identified and verified by quantitative PCR between HCC and matched normal samples. Function enrichment analysis revealed that they were associated with the wound healing process. The two lncRNAs were subsequently used to construct a prognostic risk model for HCC. Patients with high-risk scores estimated by the model showed a shorter survival time than low-risk patients (*P* < 0.001). Besides, the two lncRNA-based HCC diagnostic models exhibited good performance in discriminating HCC from normal samples on both training and test sets. The values of area under the curve (AUC) for early (I–II) and late (III–IV) HCC detection were 0.88 and 0.93, respectively.

**Conclusions:**

The two wound healing-related DElncRNAs showed robust performance for HCC prognostic prediction and detection, implying their potential role as diagnostic and prognostic markers for HCC.

## 1. Introduction

Hepatocellular carcinoma (HCC) is one of the major human malignancies and ranks as the second leading cause of cancer‐related deaths worldwide [1]. Over the past few decades, although progress in surgical techniques and systemic treatments have improved the overall prognosis of patients with liver cancer, the incidence rate continues to increase and clinical outcome is extremely dismal [2]. It is estimated that half of the worldwide new liver cancer cases and deaths occurred in China every year [3, 4]. Multiple factors including hepatitis C (HCV) and B virus (HBV) infection, toxins (aflatoxin B1), chronic alcohol abuse, and nonalcoholic fatty liver disease have been identified as risk causes of HCC [5, 6]. The classic prognostic model, tumor‐node‐metastasis (TNM) staging, as well as molecular biomarkers such as serum alpha-fetoprotein (AFP) levels, have been utilized for the diagnosis of HCC and for predicting its prognostic outcome to therapy [7]. However, due to its high heterogeneity, the prognosis and response to chemotherapy differ largely among patients with a similar stage [8]. Therefore, the search for effective biomarkers for early diagnosis and prognosis is indispensable.

lncRNAs are a class of noncoding RNAs with a length longer than 200 nucleotides (nt) that show little or no protein-coding capacity [9, 10]. In recent years, emerging studies have indicated the crucial roles of lncRNAs in pathological processes or tumorigenesis and metastasis of various cancers [11–13]. Some dysregulated lncRNAs can activate key oncogenic networks, such as the activation of epithelial-to-mesenchymal transition, Wnt, and TGF-*β* signaling pathways, promoting cancer metastasis [14]. For example, silencing of MALAT-1 by siRNA decreases cell proliferation and inhibits HCC migration and invasion [15]. Increased expression of GAS5 downregulates the vimentin and upregulates the E-cadherin level in HCC cells [16]. Given their important function in tumor development, lncRNAs as prognostic signatures in multiple cancers including renal cancer, glioblastoma, colorectal cancer, lymphoma, and others have been explored in previous studies [17–21]. To date, a variety of lncRNAs or lncRNA groups for HCC prognosis prediction have been uncovered by multiple studies [21–26]. While to the best of our knowledge, many lncRNA signatures were developed only from a single source of the dataset and few of them were both for HCC prediction of diagnosis and prognosis. So it is necessary to identify new lncRNA signatures for the early diagnosis and prognosis of HCC.

The public databases of The Cancer Genome Atlas (TCGA) and Gene Expression Omnibus (GEO) containing abundant cancer data allow researchers to exploit the potential tumorigenesis mechanisms, novel molecular subtypes, and prognostic biomarkers for various cancers [27–31]. In the current study, two novel lncRNAs were identified from two independent datasets based on our re-annotation method. Then prognostic risk models, as well as prediction diagnostic models were constructed with the two lncRNAs expression profile. We further assessed the relationship of 2-lncRNAs signature with clinicopathological parameters of the stage, grade, age, and molecular subtypes. In addition, functional enrichment analysis revealed the potential roles of 2 lncRANs in biological processes and pathways. These results indicated better performance of our 2-lncRNA signature for both the diagnostic and prognostic prediction of HCC.

## 2. Materials and Methods

### 2.1. Sample Collection and Data Preparation

We collected 14 HCC and adjacent normal FFPE samples from the Department of Oncology I of Seventh People's Hospital of Shanghai University of Traditional Chinese Medicine. The study was approved by the hospital ethics committee (No. KY2020106) and patients have been informed of the purpose of collected samples. All information regarding patient privacy has been anonymized. The dataset consisted of 11 male and 3 female patients, with a median age of 57 (Table 1).

The expression values of lncRNA based on the Reads Per Kilobases per Million (RPKM) mapped reads as well as the phenotypes and prognostic data were downloaded from the TCGA Liver Hepatocellular Carcinoma (LIHC) database (https://portal.gdc.cancer.gHCC/). Two independent expression data, GSE70880 [32] and GSE101728 [33], were downloaded from the GEO database (https://www.ncbi.nlm.nih.gov/geo/), which included 7 and 16 normal and tumor paired samples, respectively. We selected these two datasets in our study because both of them were lncRNA + mRNA microarray platforms. Additionally, the GSE144269 dataset [34] was retrieved as an independent testing set, which consisted of 140 RNA-seq samples from 70 HCC tumors and matched normal tissues. The information on the platforms and numbers of samples of each dataset were provided in Supplemental Table 1.

### 2.2. Re-Annotation of lncRNAs

The pipeline for re-annotation of lncRNA microarray probes relies on a custom Perl script [35] and the sequence alignment program of BLAT [36]. Probe sequences provided by Agilent (https://www.agilent.com/) were BLATed against the latest noncoding RNA sequences from Ensembl (https://asia.ensembl.org/Homo_sapiens/Info/Index) database with the parameter “-t = DNA -q = DNA -maxGap = 0 -out = blast8 -fastMap.” The noncoding gene alignment reports were then parsed and only the best probe-lncRNA alignment entries were kept. In order to check the consistency and detect misalignments of probes, the re-annotated probes were then compared with the probe annotation details obtained from GPL21047.

### 2.3. Differential Expression Analysis

Two independent datasets of GSE70880 and GSE101728 downloaded from the GEO database were used for the identification of differentially expressed lncRNAs (DElncRNAs) between the tumor and paired normal samples. To define the DElncRNAs, the |log2 fold change| > 1 and *P* < 0.05 were set as the threshold by using the limma R package [37]. The similarity between tumor and normal samples was evaluated by using an affinity propagation (AP) clustering algorithm [38].

### 2.4. Prognostic Risk Model Construction

Univariate Cox analysis using R survival package (https://CRAN.R-project.org/package=survival) was conducted for the identification of prognosis-associated DElncRNAs with overall survival (OS) in the TCGA HCC training set (Table 2). DElncRNAs with log-rank test *P* < 0.05 was considered as seed lncRNAs for Cox LASSO [39] regression with 10‐fold cross‐validation (CV). By 1000 iterations of Cox LASSO regression with 10‐fold CV using the R package glmnet (with the default parameter), the shrunken lncRNAs with nonzero coefficients were selected as potential prognostic lncRNAs. Multivariate Cox regression with 1000 times bootstrapping was further performed to calculate the contribution of the 2 DElncRNAs in survival predictions. The risk score of each patient was then evaluated based on the 2 DElncRNAs expression profile which is described as follows:

(1) $$\begin{array}{c} \text{Risk score}=\sum \text{Expr}\left(\text{lncRNA}\right)*\beta , \end{array}$$

where Expr and *β* are the expression value and multivariate regression coefficient of 2 DElncRNAs, respectively. The DElncRNAs with *β* > 0 were defined as high‐risk signatures while those with *β* < 0 were defined as protective genes. The patients were divided into high (Risk-H) and low (Risk-L) risk groups according to median risk score.

### 2.5. Total RNA Extraction and Quantitative PCR

Total RNA was extracted from paraffin-embedded tumor tissue and adjacent normal tissue using the Invitrogen™ TRIzol® Reagent (catalog number: 15596026, Thermo Fisher Scientific, CN) according to the kit protocol. The RNA quality was assessed using NanoDrop 2000 (Thermo, USA). Due to the extensive degradation of nucleic acid in FFPE tissues, samples with OD260/OD280 ratio between 1.5 and 2.3 were considered validated. Reverse transcription of cDNA was conducted using Promega M-MLV reverse transcriptase as described in the instructions. Quantitative PCR (qPCR) was performed on CFX96 Touch Real-Time PCR Detection System with a 20 *μ*L reaction system. Primers for GAPDH, DYNLL1-AS1, and RP11-116D2.1 are shown in Table 3. The PCR procedure consisted of pre-denaturing 95°C for 2 min, followed by 45 cycles of 95°C for 30 s, 58°C for 30 s, and 72°C for 30 s. The qPCR assay was repeated three times for each gene on each sample, and the corresponding Cq values were obtained separately. The relative expression values (2^−ΔΔCq^) of DYNLL1-AS1 and RP11-116D2.1 for each repeat were calculated according to the Cq values of GAPDH. The average of the three 2^−ΔΔCq^ values was used as the relative expression values of these two genes.

### 2.6. Functional Analysis of DElncRNAs

The co-expressed mRNA with DElncRNAs was identified by performing Pearson's correlation analyses between the expression of lncRNAs and protein-coding genes based on the RNA-seq data from the TCGA HCC cohort. Protein-coding genes with a correlation coefficient >0.5 and a false positive rate <0.05 were considered as the lncRNA-related genes. Functional annotation of the lncRNA-related genes including Gene Ontology (GO) analysis and Kyoto Encyclopedia of Genes and Genomes (KEGG) pathway enrichment were conducted by the GeneCodis3 website tool [40] (https://genecodis.cnb.csic.es/). Significantly enriched categories were identified with the threshold of Hyp_*c* < 0.05 and limited to GO terms in the “Biological Process” (GOTERM-BP-DIRECT) and KEGG pathway categories.

### 2.7. Construction of HCC Diagnostic Model

Binomial logistic regression was used to develop a diagnostic model for the detection of HCC patients based on the expression profile of 2 DElncRNAs. First, all samples from the TCGA HCC cohort were randomly separated into training and validation sets, and no significant differences were found in baseline characteristics between the two groups. The diagnostic accuracy was quantified by the area under the receiver operating characteristic (ROC) curve (AUC) [41]. Finally, the Younden index identified the optimal sensitivity and specificity. To avoid the bias of randomly grouping in a single time, the bootstrapping method with 100 times resampling was used to further evaluate the accuracy of the training set and validation set.

### 2.8. Statistical Analysis

All statistical analyses were conducted using R software (version 3.6.1). Paired Student's *t*-test was used for the comparison of lncRNAs between tumor and paired normal samples. Comparisons for two groups and multiple groups were performed for continuous variables using the Wilcox rank test and Kruskal–Wallis rank test, respectively. Categorical variable independence was established using a *χ*^2^ test. The false discovery rate was calculated using BH method.

## 3. Results

### 3.1. Identification of Differentially Expressed lncRNAs

**Figure 1:**
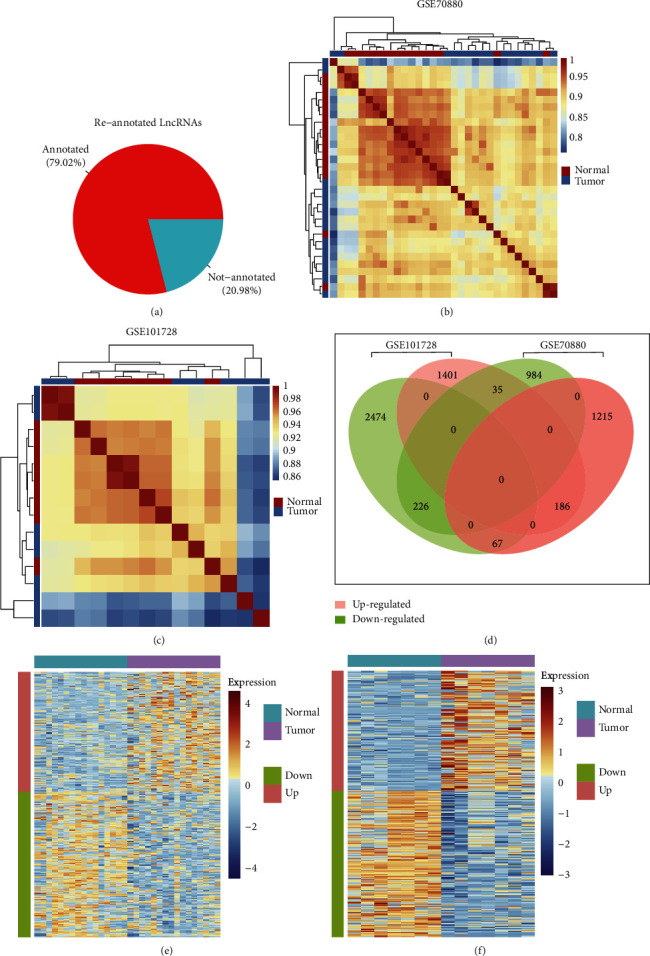
Identification of differentially expressed lncRNA. (a) Re-annotation of noncoding RNAs; (b and c) heatmap showed the distance matrix for GSE70880 (b) and GSE101728 (c); (d) comparison of differentially expressed lncRNAs between GSE70880 and GSE101728; (e and f) heatmap showed the expression of overlapped up and downregulated DElncRNAs in GSE70880 (e) and GSE101728 (f).

Our re-annotation method for the noncoding gene expression probes in GPL21047 generated 23111 best-matched lncRNAs of which 79.8% were confirmed by comparing to Agilent official data (https://www.agilent.com/) indicating high reliable expression of lncRNAs (Figure 1(a)). APClustering analysis showed systematic variations in the expression of lncRNAs and mRNAs between paired HCC and normal samples from GSE70880 and GSE101728 datasets. Samples of the two datasets were self-segregated into the HCC and normal clusters (Figures 1(b) and 1(c)). These normalized microarray expression data were used for the identification of differentially expressed lncRNAs (DElncRNAs). A total of 2713 (up/down: 1468/1245) and 4389 (up/down: 1622/2767) DElncRNAs were identified in GSE70880 and GSE101728 datasets, respectively. As for both datasets, 412 DElncRNAs (Supplementary Table 2) were shared accounting for 15.3% and 9.38% of their total DElncRNAs (Figure 1(d)). Further analysis of 412 overlapped DElncRNAs revealed that 186 DElncRNAs were significantly upregulated in HCC tissues and 226 DElncRNAs were significantly downregulated in HCC tissues (Figures 1(e) and 1(f)), indicating that the activation as well as suppression of certain biological processes regulated by these DElncRNAs potentially involved in the developing of HCC.

### 3.2. Development of HCC Prognostic Risk Model

**Figure 2:**
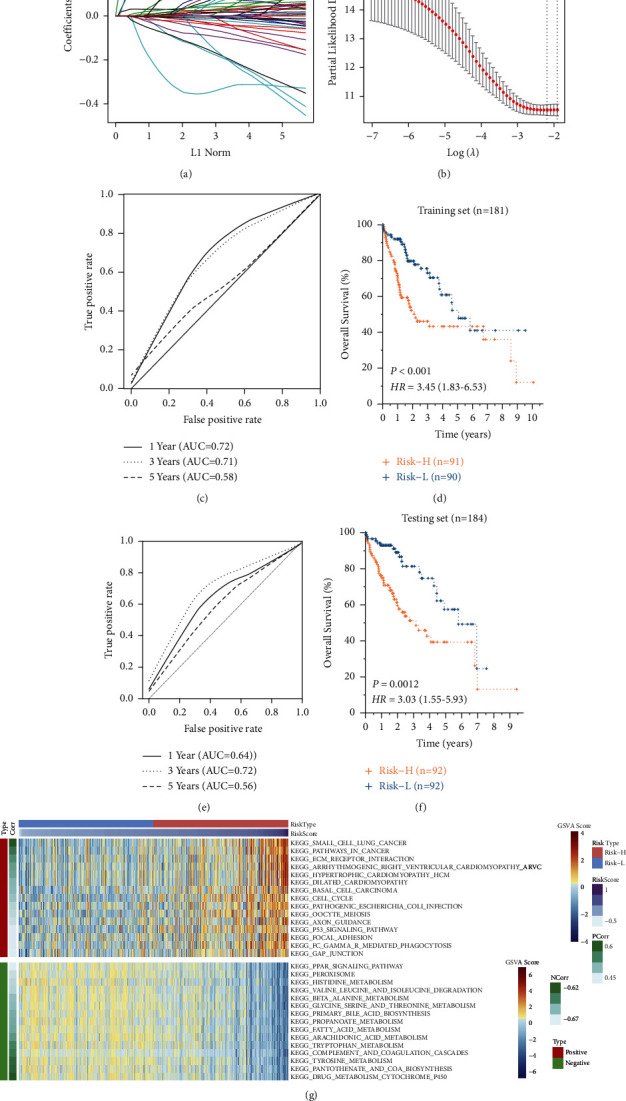
Performance of 2-lncRNA-based prognostic risk model in training and testing set. (a) 10-fold cross-validation for DElncRNAs selection in the LASSO model for OS; (b) LASSO coefficient profiles of 45 DElncRNAs for OS; (c) Time-dependent ROC analysis of 2-lncRNA risk model for predicting the overall survival of training set. The AUC was calculated for 1st, 3rd, and 5th year ROC curves; (d) Kaplan–Meier analysis for overall survival in the risk-H (*n* = 91) and risk-L (*n* = 90) groups of the training set; (e) Time-dependent ROC analysis of 2-lncRNA risk model for predicting the overall survival of testing set; (f) Kaplan–Meier analysis for overall survival in the risk-H (*n* = 92) and risk-L (*n* = 92) groups of the testing set. (g) GSVA analysis of differentially expressed genes between risk-H and risk-L group. PCorr and NCorr represent positive and negative correlations between the GSVA score and sample risk score. Heatmap showed the top 15 positive and negative correlation pathways.

The 363 TCGA HCC patients were randomly separated into training set (*n* = 181) and testing set (*n* = 182) (Table 2). Among 412 DElncRNAs identified in both in GSE70880 and GSE101728 datasets, 45 of them were detected to be also expressed in the TCGA HCC dataset. Then LASSO Cox regression method with 10‐fold cross-validation was used to construct a multivariate prognosis risk model among the 45 DElncRNAs (Figures 2(a) and 2(b)). As a result, two lncRNAs (DYNLL1-AS1 and RP11-116D2.1) remained for constructing a prognostic signature based on their expression levels and their multivariate Cox coefficients (Table 4). The performance of the 2-lncRNA signature was evaluated by a time-dependent ROC curve with the area under the curve (AUC) of 0.72, 0.71, and 0.58 for 1-, 3-, and 5-year overall survival prediction (Figure 2(c)). In the training set, HCC patients were divided into a high-risk group (Risk-H, *n* = 91) and a low-risk group (Risk-L, *n* = 90) according to the median risk score. Kaplan–Meier analysis revealed that the Risk-H group had a significantly poorer prognosis than the Risk-L group (*P* < 0.001, HR = 3.46) (Figure 2(d)). For the testing set, patients were also divided into a Risk-H (*n* = 92) and Risk-L group (*n* = 92) by using the same risk score model and cutoff value in the training set. The AUC for predicting 3-year overall survival reached 0.715 and Kaplan–Meier analysis also revealed a differential prognosis between the two groups (*P*=0.0012 and HR = 3.03) (Figures 2(e) and 2(f)), which suggested that the 2-lncRNA signature had a good performance in the prediction of 3‐year overall survival. Gene set variation analysis (GSVA) for Risk-H and Risk-L groups showed that cancer-related pathways such as cell cycle, P53 signaling pathway, and pathways in cancer had a significantly positive correlation with risk score (Figure 2(g)). While in contrast, metabolic pathways such as histidine metabolism, beta-alanine metabolism, and primary bile acid biosynthesis had significant negative correlations with risk scores (Figure 2(g)).

### 3.3. Functional Analysis of the Two lncRNAs

**Figure 3:**
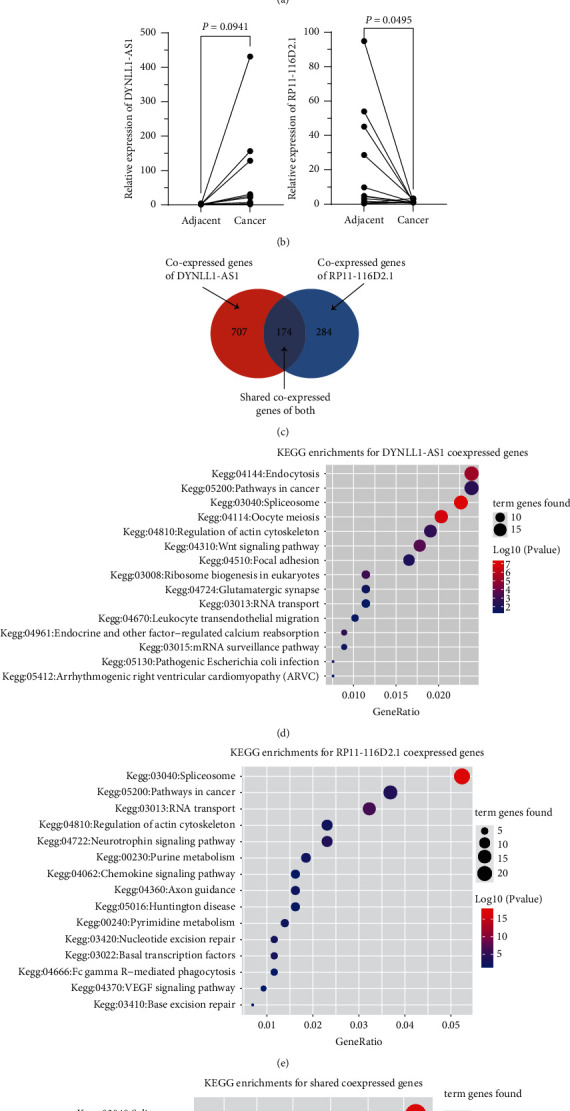
Function analysis of the 2 lncRNAs related genes. (a–c) Expression profile of DYNLL1-AS1 and RP11-116D2.1 in TCGA HCC dataset (a), GSE70880 (b), and GSE101728 (c); (d) comparison of DYNLL1-AS1- and RP11-116D2.1-related genes; (e–g) KEGG pathway enrichment of DYNLL1-AS1-related genes (e), RP11-116D2.1-related genes (f), and the overlapped-related genes (g).

The expression profile of the two lncRNAs was then evaluated in tumor and paired normal tissues from TCGA HCC and our inner HCC datasets. The results indicated that DYNLL1-AS1 was significantly upregulated and RP11-116D2.1 was significantly downregulated in the TCGA dataset (Figure 3(a)). We observed similar expression trends for these two genes between 14 HCC and adjacent normal samples, although no significant difference was found in DYNLL1-AS1 (Figure 3(b), Supplementary Table 3). Co-expression analysis identified 881 DYNLL1-AS1-related genes and 458 RP11-116D2.1-related genes, of which 174 protein-coding genes were overlapped (Figure 3(c)). KEGG pathway enrichment analysis suggested that DYNLL1-AS1-related genes are significantly enriched in pathways in cancer, focal adhesion, and the Wnt signaling pathway (Figure 3(d)). For RP11-116D2.1-related genes, they were also involved in pathways in cancer, as well as spliceosome, and RNA transport (Figure 3(e)). The overlapped significantly enriched pathways between them were spliceosome, cell cycle, oocyte meiosis, and lysine degradation (Figure 3(f)) which indicated potential interactions among the two lncRNAs.

### 3.4. Correlation of 2-lncRNA Prognostic Risk Model with Clinical Characteristics

**Figure 4:**
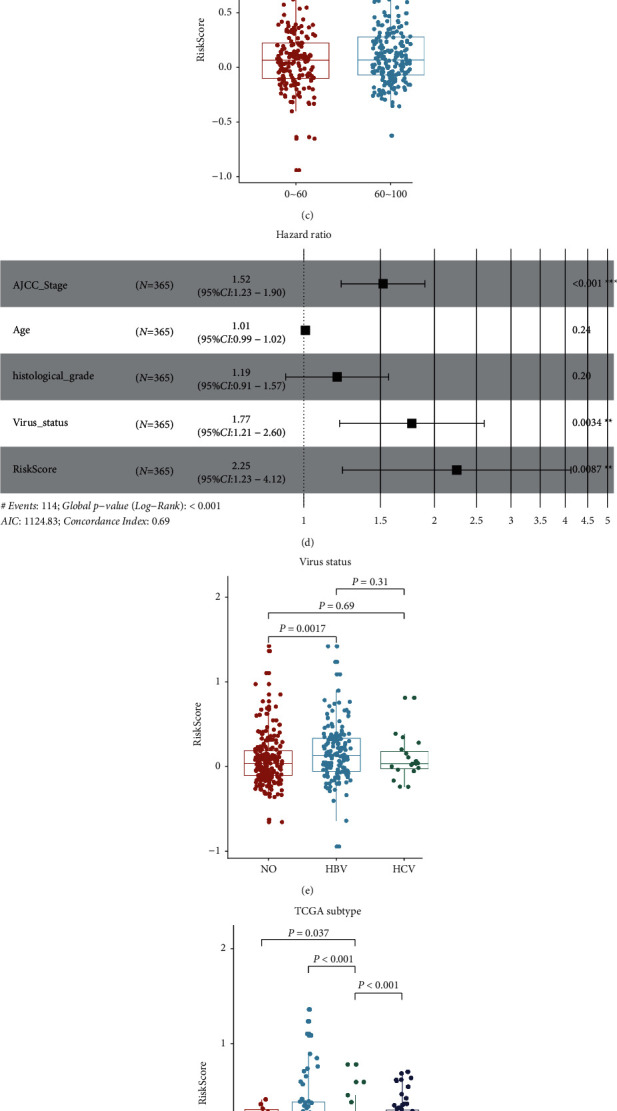
Stratification analyses of the prognostic value of the 2-lncRNA risk score for all HCC patients in the entire set. (a) Kaplan–Meier survival curve analysis of the overall survival of patients in different pathological stages; (b) Kaplan–Meier survival curve analysis of the overall survival of patients in different histological grades; (c) Scatter plot showing the correlation of age with risk scores; (d) Forest plot displaying multivariate Cox analysis of AJCC stage, age, histological grade, virus status, and risk score in the entire data set. CI, confidence interval; HR, hazard ratio; virus status, status of non-infected patients and HBV- or HCV-infected patients were assigned 0 and 1, respectively. (e–g) Boxplots illustrating the risk score of patients with different virus infections (e), different TCGA subtypes (f), and immune subtypes (g). In the boxplot, the upper and lower hinge and the inner line indicate the first and third quartile and the median value of the risk score, respectively.

**Figure 5:**
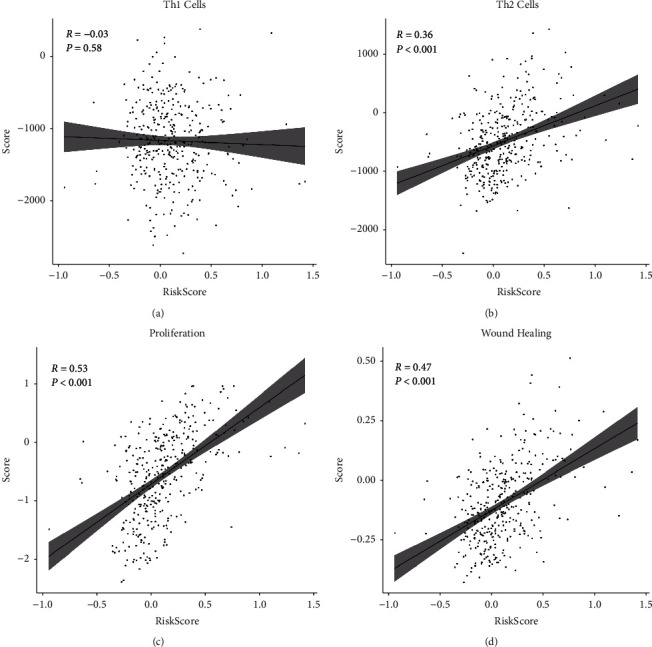
Scatterplot shows the correlation of 2-lncRNA risk scores with immune subtype scores: Th1 cells (a), The2 cells (b), proliferation (c), wound healing (d). The correlation coefficient was computed with Pearson's method.

Further analyses were performed to determine the association of the 2-lncRNA-based risk score with clinical-pathological factors in the TCGA dataset, including pathologic stage, histological grade, and age. Kaplan–Meier analysis showed a significantly different prognosis between Risk-H and Risk-L groups classified by AJCC stage I and Grade 1 and 3–4 (*P* < 0.05), indicating that the 2-lncRNA-based risk model was an independent prognostic factor of an early stage and grade (Figures 4(a) and 4(b)). Patient age also showed no significant correlation with risk scores either in Risk-H or Risk-L group (Figure 4(c)). Multivariate Cox regression analysis confirmed that the 2-lncRNA prognostic risk score was the most important hazard factor for HCC (HR = 2.25, 95%CI = 1.23–4.12, *P* < 0.001) (Figure 4(d)). Considering the infection of the virus has a great impact on HCC, we further compared the patient risk scores in HBV-infected, HCV-infected, and non-infected groups and found that the risk scores of HBV-infected samples were significantly much higher than HCV-infected and non-infected groups (Figure 4(e)). Based on the molecular level survival analysis, the LIHC cohort of the TCGA project was clustered into three molecular subtypes [42], we found that the patients in iCluster (iC) 2 with the best prognosis showed significantly lower risk scores than the patients in other two subtypes, meanwhile, the patients in iCluster 1 with the worst prognosis had the highest risk scores (Figure 4(f)). Similar results were observed among the patients from different immune subtypes. Patients in immune subtype C1 with the worst prognosis had the highest risk scores than other immune subtype patients (Figure 4(g)). The immune subtype C1 was characterized by the immune module of wound healing [43]. Given that high-risk score was mostly distributed in C1, we examined the relation between C1 signatures (wound healing-related signatures including Th1 cells, Th2 cells, proliferation, and wound healing) and risk score. Besides Th1 cells, the other three signatures were all positively correlated with risk scores (*P* < 0.01, Figure 5).

### 3.5. Development of HCC Diagnostic Model Based on the 2-lncRNA Signature

**Figure 6:**
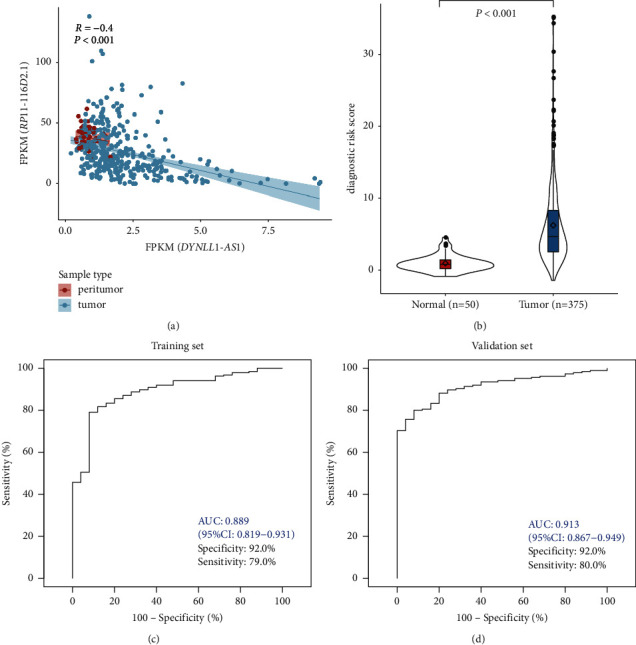
The performance of the lncRNA-based diagnostic model for HCC detection in TCGA dataset. (a) The correlation of the expression profiles of DYNLL1-AS1 and RP11-116D2.1; (b) the predicted risk scores of normal and tumor samples; (c and d) ROC curve of the dRS model in training (c) and validation (d) sets.

The logistic regression method was performed to establish a diagnostic risk score (dRS) model with the 2 lncRNAs. The expression profile of 2 lncRNAs showed that the expression of DYNLL1-AS1 was much lower than RP11-116D2.1 and their expression represented a significantly negative correlation (Figure 6(a)) suggesting a complemental expression pattern of the 2 lncRNAs. The violin plot (Figure 6(b)) showed that the dRS value was significantly upregulated in HCC tissues in the entire patient cohort. We further evaluated the performance of dRS model in discriminating HCC patients from normal controls. The ROC curve for training set and validation set was plotted (Figures 6(c) and 6(d)) with AUC of 0.889 (95%CI: 0.819–0.931) and 0.913 (95%CI: 0.867–0.949), respectively. For samples of different stages, the sensitivities were 77.19%, 80.23%, 82.35%, and 100.00% for I, II, III, and IV, respectively (Table 5).

### 3.6. Validation of the Diagnostic Model in Independent Testing Set

**Figure 7:**
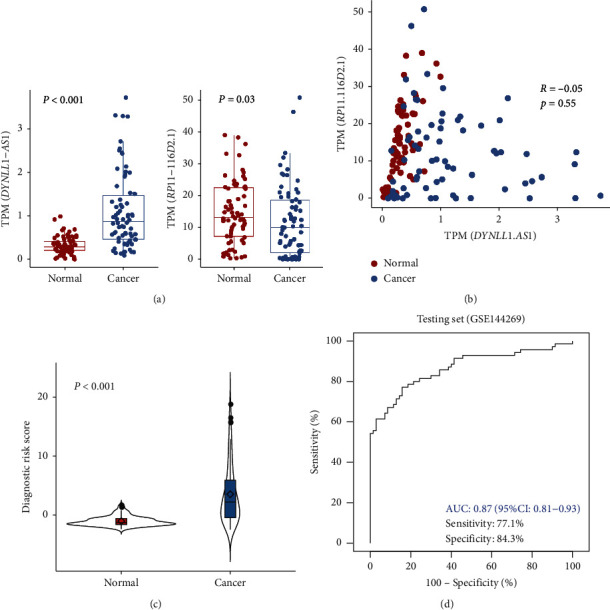
The performance of the lncRNA-based diagnostic model for HCC detection in GSE144269 independent dataset. (a) Boxplot showed the expression profiles of DYNLL1-AS1 and RP11-116D2.1 in normal and tumor samples of GSE144269; (b) the correlation of the expression profiles of DYNLL1-AS1 and RP11-116D2.1; (c) the predicted risk scores of normal and tumor samples; (d) ROC curve of the dRS model in the independent testing set.

The GSE144269 dataset containing 70 HCC tumor tissues and paired normal samples was used as a testing set to evaluate the performance of dRS model. The expression of DYNLL1-AS1 was upregulated in cancer samples, while RP11-116D2.1 showed an opposite trend (Figure 7(a)). Correlation analysis indicated that they were negatively correlated with each other (Figure 7(b)), which was consistent with the results of the TCGA dataset. The risk scores of cancer samples estimated by the model were significantly higher than that of normal samples (Figure 7(c)). ROC curve analysis showed that the model achieved an AUC of 0.87 (95% CI: 0.81–0.93) with the optimal sensitivity and specificity of 77.1% and 84.3%, respectively, as determined by the maximized Youden index (Figure 7(d)).

## 4. Discussion

Over the past decades, great efforts have been made in developing signatures for the prognostic prediction of HCC. However, no biomarkers have been shown to effectively predict the survival of HCC patients to date, partly due to its high heterogeneity causes such as virus infection, alcohol consumption, as well as immune disorders [44, 45]. Given the high morbidity and mortality of HCC, it is crucial and urgently needed to develop effective biomarkers for the prognosis prediction of HCC. Several studies have shown important implications of molecular biomarkers such as aberrantly expressed genes and abnormal methylation events for outcome prediction and therapy decisions [17, 20, 46, 47]. Recently, lncRNAs were reported as critical regulators in various diseases including cancers [12, 13]. Moreover, their potential utility as prognostic biomarkers for HCC was also demonstrated in a few studies [18, 21].

The current study identified a series of differentially expressed lncRNAs by integrating two independent gene expression microarray datasets, among which 412 DElncRNAs were shared by the two datasets. Two lncRNAs were subsequently selected by performing LASSO regression with 10‐fold cross‐validation (CV). Results of multiple datasets indicated that expressions of DYNLL1-AS1 were significantly upregulated in cancer cells, while it was the opposite for RP11-116D2.1. We also observed a similar tendency in our custom dataset. Notably, no significant difference in RP11-116D2.1 expression was found in our dataset (*P*=0.094), which we supposed might be related to several reasons. Firstly, the small sample size could lead to errors when using a two-sample *t*-test, as only 14 samples were quantified in this study, and the power was only 0.93 for paired *t*-test, which is slightly lower than 0.95 (*n* = 14). According to our clinical data (Table 1), most of the samples were stage II, which leads to a more unbalanced distribution of clinical stages compared with the TCGA dataset. Secondly, the degradation, fragmentation, and chemical modification of nucleic acid frequently occur in FFPE tissues, which will affect the quantification of gene expression [48]. Nevertheless, our results showed a similar trend of upregulated DYNLL1-AS1 in HCC samples, and the effects caused by FFPE samples can be eliminated by the relative quantification base on the internal reference gene, making this result reliable to some extent.

Survival analysis indicated that patients with higher expressions of DYNLL1-AS1 were found to correlate with shorter survival time, while the elevated expressions of RP11-116D2.1 were associated with longer survival time. The two lncRNAs were then used to construct a prognostic prediction model for HCC. ROC curve analysis suggested that the model was robust for HCC survival prediction. The 2-lncRNA signature showed a higher AUC value than the 5-lncRNA signature [49] and nearly the same AUC as 7-lncRNA signature [50]. Obviously, it is more feasible for researchers to carry out further investigations of less lncRNAs. These results revealed that the two lncRNAs could serve as promising prognostic factors for HCC.

Previous studies have revealed that HBV infection was a risk factor for HCC prognosis [51]. A significantly higher risk score estimated by this model was also observed in the HBV-infected group than in the non-infected group. Besides, the prognostic model based on lncRNAs developed by Xiwen Liao et al. showed a good performance in HBV-related HCC [52], which was comparable with our model in terms of AUC values. Our findings implied their potential association with HBV infection.

Functional enrichment analysis indicated that genes correlated with DYNLL1-AS1 and RP11-116D2.1 significantly enriched in the Wnt signaling pathway, chemokine signaling pathway, and VEGF signaling pathway. Interestingly, we observed a significant correlation between the 2-lncRNA signature and wound healing subtype (C1) of HCC. The wound healing response involves several phases, including the formation of a fibrin clot at the wound site and the infiltration of neutrophils in the early stage, where these immune cells can release a plethora of cytokines and chemokines [53]. Another critical phase is epithelial regeneration, which relates to the migration and proliferation of fibroblasts [54]. Additionally, ECM remodeling is another important event in wound healing [55]. In this study, the risk score estimated by the 2-lncRNA signature showed a strong correlation with the enriched score of the ECM receptor pathway. These findings suggested critical roles of the two lncRNAs in inflammatory pathways, however, more robust experiments are needed to investigate their potential relationship with the inflammatory response.

The testing and abdominal ultrasound of serum biomarker alpha-fetoprotein (AFP) were widely recommended for routine surveillance of HCC in high-risk patients (US) according to many HCC guidelines [56], however, it has been excluded from the surveillance and diagnosis criteria in the guidelines published in 2014 [57]. Other serum biomarkers such as AFP-L3, DCP, interleukin-6, interleukin-10, and squamous cell carcinoma antigen were also investigated, while these serum-based tests lack adequate sensitivity and specificity for effective surveillance [58–60]. In this study, we further developed a model for HCC detection based on the two lncRNA expression profiles. The performance of the 2-lncRNA diagnostic model achieved AUC values of 0.889 and 0.913 in the training and validation sets, respectively, which was better than the efficiency of serum biomarkers. Moreover, we also obtained an AUC of 0.87 (95%CI: 0.81–0.93) in the independent testing set of GSE144269. For early stage (I–II) HCC, the diagnostic model achieved an AUC of 0.88. Previous studies have demonstrated the favorable efficiency of lncRNAs for HCC detection. Our results showed a comparable performance relative to other promising biomarkers such as DANCR [61], HULC [62], and Linc00152 [63], suggesting that two lncRNAs could be promising candidates in diagnosing ontogenesis of HCC.

## 5. Conclusions

In this study, we identified two differentially expressed lncRNAs from multiple datasets verified their expressions in our custom HCC samples. The 2-lncRNA signature showed robust performance for HCC detection and prognosis prediction. However, some limitations of the study need to be considered. For example, further experiments should be carried out to demonstrate their potential roles in HBV infection as well as their relationship with wound healing. Their performance for HCC detection and prognosis prediction needs to be evaluated in more clinical samples. Besides, the exact mechanisms of the two lncRNAs in HCC tumorigenesis and progression are still not well-studied although this study has comprehensively revealed their aberrant expressions between normal and cancer samples.

## Figures and Tables

**Table 1:** Clinicopathological features of 14 patients.

Patient ID	Sample ID	Age	Sex	Metastasis	Grade	Tumor location	AJCC stage
H1	HCC1	57	Male	No	2	Left-sided	I
H2	HCC2	58	Female	No	2	Right-sided	II
H3	HCC3	67	Male	No	2	Right-sided	I
H4	HCC4	66	Female	No	2	Right-sided	I
H5	HCC5	56	Male	No	2	Right-sided	II
H6	HCC6	62	Male	No	2	Right-sided	II
H7	HCC7	51	Male	No	2	Right-sided	II
H8	HCC8	52	Male	No	2	Left-sided	II
H9	HCC9	55	Male	No	2	Right-sided	II
H10	HCC10	51	Male	No	2	Right-sided	II
H11	HCC11	70	Male	No	2	Left-sided	II
H12	HCC12	NA	Male	No	3	Left-sided	II
H13	HCC13	72	Male	No	3	Right-sided	I
H14	HCC14	44	Female	No	3	Left-sided	IV

**Table 2:** Clinical features of HCC patients in training and testing set.

Clinical features	Training set	Testing set	*P* value
Event			0.23
Alive	111	124	
Dead	70	60	

Gender			0.37
Female	55	64	
Male	126	120	

Pathological stage			0.14
Stage I	97	73	
Stage II	35	49	
Stage III	2	1	
Stage IIIA	28	35	
Stage IIIB	3	5	
Stage IIIC	3	6	
Stage IV	1	3	
Not available	12	2	

Histological grade			0.44
G1	31	24	
G2	79	96	
G3	59	59	
G4	7	5	
Not available	5	0	

*P* value was calculated by the Fisher-exact test.

**Table 3:** Primers of DYNLL1-AS1 and RP11-116D2.1 used for quantitative PCR.

Primer	Sequence (5′->3′)
GAPDH F	GGACTCATGACCACAGTCCA
GAPDH R	TCAGCTCAGGGATGACCTTG
DYNLL1-AS1 F	CCAGCTGTCTGGAGAGATGAA
DYNLL1-AS1 R	TCGGAGGCATCAACTCCTTT
RP11-116D2.1 F	ATGGGTGGGTGAGCGAATAA
RP11-116D2.1 R	TCCAGGCCTCCTTTCAGTTT

**Table 4:** Details of the two lncRNA signatures.

hg38 name	Ensembl_ID	TransID	FC (T/N)	Regulation	*P* Value	Coefficient	HR
DYNLL1-AS1	ENSG00000248008.2	ENST00000500741.2	1.246	Up	0.019	0.1514	1.18
RP11-116D2.1	ENSG00000261012.2	ENST00000567376.2	0.901	Down	0.0066	-0.0078	0.98

FC: fold change; T/N: tumor/normal; HR: hazard ratio.

**Table 5:** Performance of 2-lncRNA signature for the detection of HCC patients with different stages.

	Stage I	Stage II	Stage III	Stage IV	Other	Total
AUC	0.86	0.91	0.93	0.96	0.93	0.89
Sensitivity	77.19%	80.23%	82.35%	100.00%	91.67%	76.55%
Specificity	86.00%	92.00%	92.00%	80.00%	86.00%	92.00%

## Data Availability

Data from cancer patients used in this study were collected from the publicly available de-identified hepatocellular carcinoma data set from Gene Expression Omnibus (GEO) and The Cancer Genome Atlas (TCGA). All the intermediate data are supplied in this manuscript and the supplementary materials.

## Supplementary Materials

Supplementary Materials Supplementary Table 1. Datasets used in this study. Supplementary Table 2. The overlapped differentially expressed lncRNAs between GSE70880 and GSE101728. Supplementary Table 3. The relative expressions of DYNLL1-AS1 and RP11-116D2.1 between 14 HCC and adjacent normal samples.
