# Supplementary material for: Identification of a Two-lncRNA Signature with Prognostic and Diagnostic Value for Hepatocellular Carcinoma
Source: J Oncol. 2022 Jul 21;2022:2687455. doi: 10.1155/2022/2687455 (PMC9546683; doi:10.1155/2022/2687455)
Supplement: Supplementary Materials — Supplementary Table 1. Datasets used in this study. Supplementary Table 2. The overlapped differentially expressed lncRNAs between GSE70880 and GSE101728. Supplementary Table 3. The relative expressions of DYNLL1-AS1 and RP11-116D2.1 between 14 HCC and adjacent normal samples. [file 2687455.f1.docx]

**Supplementary tables**

**Supplementary Table 1 Datasets used in this study.**

|  | Normal | Tumor | Platform | Description |
| --- | --- | --- | --- | --- |
| TCGA | 50 | 371 | GPL16791 | RNA-seq |
| GSE70880 | 16 | 16 | GPL19748 | Agilent-038314 CBC Homo sapiens lncRNA + mRNA microarray V2.0 |
| GSE101728 | 7 | 7 | GPL21047 | Agilent-074348 Human LncRNA v6 4X180K |
| GSE144269 | 70 | 70 | GPL24676 | RNA-seq |

**Supplementary Table 2 The overlapped differentially expressed lncRNAs between GSE70880 and GSE101728.**

| gName | Probe | GSE70880_fc | GSE70880_p | GSE101728_fc | GSE101728_p | Direction |
| --- | --- | --- | --- | --- | --- | --- |
| DLEU2 | ENST00000235290.7 | 1.0476 | 0.041 | 1.8469 | 0.0014 | Up |
| LINC01116 | ENST00000295549.9 | 1.1871 | 0.0099 | 1.3463 | 0.0064 | Up |
| LINC00471 | ENST00000313064.3 | 1.1314 | 0.0089 | 1.4643 | 0.0202 | Up |
| TYMSOS | ENST00000323813.3 | 1.2811 | 1.00E-04 | 1.1457 | 4.00E-04 | Up |
| CYTOR | ENST00000331944.10 | 1.0855 | 0.0113 | 1.3035 | 4.00E-04 | Up |
| KTN1-AS1 | ENST00000335142.5 | 1.1407 | 0.0227 | 1.1633 | 0.0037 | Up |
| AC010442.1 | ENST00000342584.3 | 1.0861 | 1.00E-04 | 1.3037 | 0.0041 | Up |
| NBR2 | ENST00000356906.7 | 1.0337 | 0.0179 | 1.1142 | 0.0056 | Up |
| NPSR1-AS1 | ENST00000358772.8 | 1.3399 | 0.0078 | 1.587 | 0.006 | Up |
| SNORA74A | ENST00000364089.1 | 1.0787 | 0.011 | 1.2757 | 0.0023 | Up |
| LINC01270 | ENST00000371639.8 | 1.0723 | 5.00E-04 | 1.1681 | 0.0038 | Up |
| SNHG32 | ENST00000375633.5 | 1.0667 | 0.0013 | 1.0629 | 0.0483 | Up |
| AC006305.1 | ENST00000382897.2 | 1.2415 | 0.0061 | 1.4301 | 0.0011 | Up |
| TDRKH-AS1 | ENST00000389897.3 | 1.0781 | 0.0074 | 1.7876 | 7.00E-04 | Up |
| APTR | ENST00000398043.3 | 1.0468 | 0.0352 | 1.1691 | 7.00E-04 | Up |
| CYTOR | ENST00000409054.2 | 1.1578 | 1.00E-04 | 1.3046 | 0.0065 | Up |
| CYTOR | ENST00000409139.6 | 1.1326 | 3.00E-04 | 1.3233 | 0.0066 | Up |
| RNU4-25P | ENST00000410569.1 | 1.1535 | 0.0479 | 1.4062 | 0.0376 | Up |
| RN7SKP121 | ENST00000410842.1 | 1.061 | 0.0078 | 1.425 | 0.0064 | Up |
| AC003975.1 | ENST00000411856.1 | 1.1266 | 0.0354 | 1.0983 | 0.0216 | Up |
| LNCTAM34A | ENST00000412639.3 | 1.042 | 0.0131 | 1.2297 | 0.0121 | Up |
| CR759835.1 | ENST00000412771.1 | 1.1821 | 0.0012 | 1.3198 | 0.017 | Up |
| SLC16A1-AS1 | ENST00000413231.5 | 1.1275 | 0.0077 | 1.154 | 0.0367 | Up |
| SPATA3-AS1 | ENST00000414876.5 | 1.0365 | 0.0284 | 1.0842 | 0.013 | Up |
| AL731684.1 | ENST00000415530.1 | 1.1806 | 2.00E-04 | 2.9772 | 0.0047 | Up |
| HCG18 | ENST00000415873.5 | 1.0622 | 0.0088 | 1.2037 | 0.0147 | Up |
| AL590705.3 | ENST00000416066.1 | 1.1064 | 0 | 1.1602 | 0 | Up |
| GTF3C2-AS1 | ENST00000416453.7 | 1.1457 | 3.00E-04 | 1.0605 | 0.035 | Up |
| Z98257.1 | ENST00000417884.1 | 1.0464 | 7.00E-04 | 1.4934 | 0.0266 | Up |
| LINC02542 | ENST00000418567.2 | 1.1596 | 0.0176 | 1.2246 | 0.0424 | Up |
| AL162582.1 | ENST00000419196.1 | 1.2625 | 0.0011 | 1.2736 | 0.0182 | Up |
| AL121999.1 | ENST00000420156.2 | 1.1327 | 0.0229 | 1.2088 | 0.0179 | Up |
| AL392172.1 | ENST00000420335.5 | 1.0664 | 3.00E-04 | 1.0742 | 0.0342 | Up |
| KCNMB2-AS1 | ENST00000421498.1 | 1.0693 | 0.0426 | 2.3574 | 0.0174 | Up |
| CDKN2B-AS1 | ENST00000421632.2 | 1.256 | 0.0084 | 2.1377 | 9.00E-04 | Up |
| TMCO1-AS1 | ENST00000423121.1 | 1.1961 | 5.00E-04 | 1.384 | 0.0117 | Up |
| AL391832.1 | ENST00000423963.1 | 1.1413 | 0.0279 | 1.935 | 0.0065 | Up |
| AL354824.1 | ENST00000424931.1 | 1.1247 | 0.047 | 1.681 | 0.0346 | Up |
| UBAC2-AS1 | ENST00000426037.6 | 1.1367 | 0.0181 | 1.1652 | 0.034 | Up |
| AC007405.3 | ENST00000426475.2 | 1.1687 | 0.015 | 1.2191 | 0.0133 | Up |
| HAGLROS | ENST00000426615.3 | 1.268 | 0 | 2.4598 | 0.0075 | Up |
| RNASEH1-AS1 | ENST00000426725.1 | 1.1015 | 4.00E-04 | 1.0963 | 0.0187 | Up |
| AL672310.1 | ENST00000426839.2 | 1.0522 | 0.0045 | 1.398 | 0.0107 | Up |
| LINC00708 | ENST00000428165.1 | 1.039 | 0.005 | 1.1505 | 0.0225 | Up |
| MAPKAPK5-AS1 | ENST00000428207.4 | 1.057 | 0.0111 | 1.0737 | 0.0125 | Up |
| HMGA1P4 | ENST00000428643.1 | 1.0811 | 0.002 | 1.1178 | 0.0162 | Up |
| AL133216.1 | ENST00000428915.6 | 1.0321 | 0.0079 | 1.0912 | 0.0314 | Up |
| XIST | ENST00000429829.6 | 1.0394 | 0.0028 | 1.3951 | 0.0066 | Up |
| AL078604.2 | ENST00000430078.1 | 1.0483 | 0.0352 | 1.4451 | 0.0101 | Up |
| AL356056.2 | ENST00000430188.1 | 1.1327 | 0.0197 | 1.5917 | 0.0096 | Up |
| LINC00299 | ENST00000430192.5 | 1.2384 | 0.0064 | 1.2339 | 0.0242 | Up |
| FAM41C | ENST00000432963.1 | 1.0559 | 0.0015 | 1.0579 | 0.0192 | Up |
| LINC00205 | ENST00000433465.2 | 1.1159 | 0.0194 | 1.2432 | 0.0024 | Up |
| AL390957.1 | ENST00000434300.2 | 1.3658 | 4.00E-04 | 1.237 | 0.0298 | Up |
| LINC02816 | ENST00000434447.5 | 1.0348 | 0.0385 | 1.2082 | 0.0041 | Up |
| AL118511.2 | ENST00000436739.1 | 1.0834 | 0.027 | 1.9523 | 0.0393 | Up |
| AL603839.2 | ENST00000437060.1 | 1.036 | 4.00E-04 | 1.0617 | 0.0054 | Up |
| RNASEH1-AS1 | ENST00000438436.3 | 1.1472 | 1.00E-04 | 1.338 | 0.0064 | Up |
| AL392172.1 | ENST00000439440.1 | 1.0757 | 0.0025 | 1.2901 | 0.0034 | Up |
| AL592435.1 | ENST00000439699.1 | 1.1807 | 0.0048 | 1.2629 | 0.008 | Up |
| LNCAROD | ENST00000443523.2 | 1.482 | 0.0023 | 3.7168 | 1.00E-04 | Up |
| FOXD2-AS1 | ENST00000445551.1 | 1.0952 | 0.0129 | 1.104 | 0.0221 | Up |
| RUSC1-AS1 | ENST00000446880.5 | 1.0956 | 0.025 | 1.2185 | 0.0089 | Up |
| AP001469.3 | ENST00000447037.1 | 1.0995 | 2.00E-04 | 1.2129 | 0.001 | Up |
| AC019080.1 | ENST00000447413.1 | 1.092 | 0.0206 | 1.282 | 0.0284 | Up |
| AC092650.1 | ENST00000447571.5 | 1.1124 | 0.0023 | 1.5519 | 0.0159 | Up |
| DLG5-AS1 | ENST00000449852.1 | 1.1621 | 1.00E-04 | 1.8751 | 0.0209 | Up |
| ST8SIA6-AS1 | ENST00000451225.2 | 1.3959 | 0.0033 | 2.2516 | 0.027 | Up |
| AC000067.1 | ENST00000452326.1 | 1.0844 | 0.0306 | 1.4477 | 0.0162 | Up |
| LINC02802 | ENST00000452399.6 | 1.2302 | 0 | 1.0591 | 0.0325 | Up |
| ASH1L-AS1 | ENST00000452809.1 | 1.068 | 0.001 | 1.1251 | 0.0423 | Up |
| RASAL2-AS1 | ENST00000452867.1 | 1.1042 | 0.0033 | 1.5146 | 0.0019 | Up |
| CDKN2B-AS1 | ENST00000455933.7 | 1.1826 | 0.0093 | 1.7121 | 0.001 | Up |
| ELOVL2-AS1 | ENST00000456190.6 | 1.1488 | 0.0126 | 1.6227 | 0.0427 | Up |
| LINC01923 | ENST00000456248.1 | 1.0495 | 0.0493 | 1.3407 | 0.0291 | Up |
| AC068196.1 | ENST00000456895.1 | 1.0496 | 0.0203 | 1.2553 | 0.0092 | Up |
| LINC01270 | ENST00000457853.1 | 1.0797 | 0.0055 | 1.0428 | 0.0263 | Up |
| DUBR | ENST00000463143.5 | 1.0459 | 0.0018 | 1.2006 | 8.00E-04 | Up |
| AC092910.3 | ENST00000469070.1 | 1.1028 | 0.0105 | 1.25 | 0.0256 | Up |
| AC002116.1 | ENST00000473572.2 | 1.0409 | 0.011 | 1.0813 | 0.0127 | Up |
| LINC02050 | ENST00000473938.1 | 1.1322 | 0.0043 | 1.4795 | 0.0231 | Up |
| PTPRG-AS1 | ENST00000474795.5 | 1.0702 | 0.0032 | 1.2671 | 0.0354 | Up |
| AC092910.3 | ENST00000484076.1 | 1.053 | 0.0221 | 1.2344 | 0.0388 | Up |
| AC010655.2 | ENST00000493710.1 | 1.0698 | 0.0085 | 1.2612 | 0.0347 | Up |
| ATP1A1-AS1 | ENST00000493908.2 | 1.0366 | 0.0312 | 1.0465 | 0.0493 | Up |
| AL662884.2 | ENST00000494022.1 | 1.0352 | 0.0053 | 1.0829 | 0.0072 | Up |
| DNAJC3-DT | ENST00000499499.2 | 1.0688 | 0.0135 | 1.1692 | 0.0228 | Up |
| UBR5-AS1 | ENST00000499653.1 | 1.0748 | 4.00E-04 | 1.1491 | 0.0106 | Up |
| NSMCE1-DT | ENST00000499939.2 | 1.0943 | 0.0468 | 1.1767 | 9.00E-04 | Up |
| SBF2-AS1 | ENST00000499953.6 | 1.086 | 0.0073 | 1.1886 | 0.0166 | Up |
| LINC00461 | ENST00000500197.6 | 1.0598 | 0.0334 | 1.0958 | 0.0434 | Up |
| NRAV | ENST00000500741.2 | 1.0493 | 0.0209 | 1.4432 | 0.018 | Up |
| RAB30-DT | ENST00000501011.7 | 1.0545 | 0.0015 | 1.0746 | 0.0069 | Up |
| AC006064.2 | ENST00000501075.2 | 1.0346 | 0.0046 | 1.413 | 0.0369 | Up |
| AC090061.1 | ENST00000502102.2 | 1.0893 | 0.0084 | 1.1902 | 0.0094 | Up |
| AC135457.1 | ENST00000503553.3 | 1.0305 | 0.0173 | 1.0893 | 0.0234 | Up |
| HULC | ENST00000503668.2 | 1.0811 | 0.0109 | 1.0366 | 0.004 | Up |
| AC127070.2 | ENST00000503695.4 | 1.1321 | 0.0045 | 1.4115 | 0.0129 | Up |
| CASC9 | ENST00000504531.2 | 1.4992 | 4.00E-04 | 1.3876 | 0.0075 | Up |
| AC093810.1 | ENST00000505511.1 | 1.272 | 0.009 | 1.1844 | 0.0401 | Up |
| STX18-AS1 | ENST00000507244.6 | 1.0604 | 4.00E-04 | 1.5294 | 0.0014 | Up |
| TRIM52-AS1 | ENST00000507434.1 | 1.1784 | 3.00E-04 | 1.2973 | 0.0139 | Up |
| SEPSECS-AS1 | ENST00000507794.2 | 1.0703 | 0.0372 | 1.1213 | 0.042 | Up |
| AC139493.2 | ENST00000508056.5 | 1.1254 | 0.0093 | 1.2481 | 0.0122 | Up |
| AC016550.2 | ENST00000510946.5 | 1.5296 | 0.0055 | 1.5344 | 0.0469 | Up |
| PVT1 | ENST00000512617.7 | 1.1691 | 0.0056 | 2.5079 | 2.00E-04 | Up |
| AC104446.1 | ENST00000516978.2 | 1.1288 | 4.00E-04 | 1.567 | 0.0082 | Up |
| LINC00535 | ENST00000517785.1 | 1.2038 | 0.0146 | 1.7566 | 0.0164 | Up |
| AC104109.4 | ENST00000518409.1 | 1.0693 | 0.0074 | 1.0638 | 0.0029 | Up |
| TUG1 | ENST00000519077.4 | 1.0305 | 0.0421 | 1.1355 | 0.0461 | Up |
| LINC02202 | ENST00000523301.1 | 1.1343 | 0.0025 | 1.0498 | 0.0096 | Up |
| AC009630.2 | ENST00000524133.1 | 1.2545 | 0 | 1.551 | 0.0115 | Up |
| ALG1L9P | ENST00000525473.1 | 1.1662 | 0.03 | 1.3228 | 0.0183 | Up |
| AC055860.1 | ENST00000525523.1 | 1.1023 | 0.0114 | 1.0971 | 7.00E-04 | Up |
| SBF2-AS1 | ENST00000526617.1 | 1.2019 | 0.005 | 1.1762 | 0.0239 | Up |
| AP003068.2 | ENST00000526623.2 | 1.0592 | 0.0015 | 1.1206 | 0.0062 | Up |
| AC069287.3 | ENST00000527297.1 | 1.231 | 0.0215 | 1.975 | 0.0306 | Up |
| AL136309.4 | ENST00000527831.1 | 1.101 | 0.0401 | 1.2909 | 0.0304 | Up |
| AP000873.2 | ENST00000529607.1 | 1.0381 | 0.0059 | 1.3228 | 0.0047 | Up |
| AP003086.1 | ENST00000534168.1 | 1.1297 | 0.0051 | 1.2321 | 0.0195 | Up |
| SNHG1 | ENST00000535076.6 | 1.0553 | 0.0117 | 1.092 | 0.0114 | Up |
| SNHG1 | ENST00000538266.5 | 1.0898 | 0 | 1.1861 | 0.011 | Up |
| AC092112.1 | ENST00000538329.1 | 1.1983 | 6.00E-04 | 2.4362 | 0.0261 | Up |
| AP000777.3 | ENST00000538705.1 | 1.0815 | 1.00E-04 | 1.1061 | 6.00E-04 | Up |
| SNHG1 | ENST00000539303.5 | 1.1017 | 0 | 1.2677 | 0.0106 | Up |
| AC063926.1 | ENST00000539532.1 | 1.0587 | 0.0341 | 1.3757 | 0.0281 | Up |
| KDM2B-DT | ENST00000541574.1 | 1.1625 | 0.0078 | 1.3402 | 0.02 | Up |
| GIHCG | ENST00000546580.2 | 1.1364 | 0.0011 | 1.1098 | 0.0208 | Up |
| LINC02588 | ENST00000548328.5 | 1.3391 | 0.0205 | 2.4299 | 0.0198 | Up |
| AC125611.3 | ENST00000550468.2 | 1.1042 | 0 | 1.1562 | 0.0016 | Up |
| LINC02308 | ENST00000555001.2 | 1.056 | 0.0364 | 1.1696 | 0.0385 | Up |
| SNHG21 | ENST00000558174.5 | 1.1122 | 0.0036 | 1.133 | 0.0045 | Up |
| AC104590.1 | ENST00000558368.2 | 1.168 | 0.0023 | 2.1978 | 0.0178 | Up |
| CERNA1 | ENST00000559779.2 | 1.1782 | 2.00E-04 | 1.3732 | 0.0471 | Up |
| AC100830.1 | ENST00000560387.1 | 1.09 | 0.0042 | 1.0266 | 0.0391 | Up |
| AC090517.5 | ENST00000561122.1 | 1.1066 | 7.00E-04 | 1.651 | 0.0333 | Up |
| AC111152.2 | ENST00000561392.1 | 1.0499 | 0.0039 | 2.1394 | 0.0132 | Up |
| FBXL19-AS1 | ENST00000563777.1 | 1.0918 | 1.00E-04 | 1.2969 | 0.0227 | Up |
| AC100821.2 | ENST00000565668.2 | 1.0739 | 0.0155 | 1.3508 | 0.0411 | Up |
| AC010547.1 | ENST00000567341.1 | 1.0915 | 0.0418 | 1.7886 | 0.0326 | Up |
| AC139099.2 | ENST00000574856.1 | 1.029 | 0.0458 | 1.2373 | 0.0266 | Up |
| AC139530.1 | ENST00000575312.1 | 1.0321 | 3.00E-04 | 1.175 | 0 | Up |
| AC015674.1 | ENST00000578482.1 | 1.0324 | 7.00E-04 | 1.0835 | 4.00E-04 | Up |
| AC010327.5 | ENST00000585911.1 | 1.1297 | 0.001 | 1.5323 | 9.00E-04 | Up |
| AC008735.2 | ENST00000585940.1 | 1.0346 | 5.00E-04 | 1.2491 | 0.007 | Up |
| AC011815.1 | ENST00000586922.2 | 1.1168 | 0.0108 | 1.451 | 0.0445 | Up |
| LINC01532 | ENST00000591143.6 | 1.1402 | 3.00E-04 | 1.542 | 0.0306 | Up |
| AC002094.1 | ENST00000591482.1 | 1.0412 | 0.0129 | 1.0757 | 0.0275 | Up |
| AC011446.1 | ENST00000591825.1 | 1.0602 | 0.002 | 1.0734 | 0.0474 | Up |
| AC011518.1 | ENST00000592668.2 | 1.0678 | 0.034 | 1.0742 | 0.0035 | Up |
| AC011503.1 | ENST00000596326.5 | 1.1148 | 0.0174 | 1.0598 | 0.0058 | Up |
| PTOV1-AS1 | ENST00000596521.1 | 1.0715 | 0.0146 | 1.4993 | 0.0182 | Up |
| ZSCAN16-AS1 | ENST00000600652.1 | 1.1846 | 4.00E-04 | 1.1449 | 4.00E-04 | Up |
| SMC2-AS1 | ENST00000603487.1 | 1.0652 | 0.023 | 1.2853 | 0.0309 | Up |
| AL365181.3 | ENST00000605886.1 | 1.1805 | 0.0013 | 1.9967 | 0.0082 | Up |
| AC012640.4 | ENST00000606194.1 | 1.0666 | 0.025 | 1.1227 | 0.0088 | Up |
| AL024498.1 | ENST00000606522.1 | 1.136 | 5.00E-04 | 1.7717 | 0 | Up |
| SNHG31 | ENST00000607412.1 | 1.1779 | 0.0068 | 1.025 | 0.0311 | Up |
| AC073529.1 | ENST00000608176.5 | 1.3304 | 7.00E-04 | 1.5941 | 0.0395 | Up |
| AC090114.2 | ENST00000608477.1 | 1.0845 | 0 | 1.2228 | 0.0417 | Up |
| AC024560.3 | ENST00000608482.1 | 1.0969 | 6.00E-04 | 1.4676 | 0.0194 | Up |
| ATP1A1-AS1 | ENST00000608511.6 | 1.0335 | 0.0481 | 1.1131 | 0.0055 | Up |
| AC005229.4 | ENST00000610085.1 | 1.0723 | 0.0198 | 1.1977 | 0.0194 | Up |
| AC025580.3 | ENST00000617932.1 | 1.1723 | 0.0293 | 1.8392 | 0.0102 | Up |
| AL139260.2 | ENST00000622355.1 | 1.0385 | 1.00E-04 | 1.0939 | 0.0141 | Up |
| AC024940.6 | ENST00000622889.1 | 1.0578 | 0.0455 | 1.1328 | 0.0319 | Up |
| CTBP1-AS | ENST00000625256.1 | 1.027 | 0.0086 | 1.7269 | 0.0031 | Up |
| LINC01232 | ENST00000625287.1 | 1.0486 | 0.0368 | 1.0835 | 0.0396 | Up |
| THRIL | ENST00000627370.1 | 1.0655 | 0.0014 | 1.1474 | 2.00E-04 | Up |
| LINC01138 | ENST00000639181.1 | 1.0542 | 4.00E-04 | 1.4292 | 1.00E-04 | Up |
| AC092821.3 | ENST00000641304.1 | 1.1316 | 0 | 1.4223 | 0.0044 | Up |
| MIR4435-2HG | ENST00000643013.1 | 1.1489 | 3.00E-04 | 1.0981 | 0.0483 | Up |
| AL445989.2 | ENST00000649617.1 | 1.2276 | 0.0066 | 1.612 | 0.0465 | Up |
| AC023593.1 | ENST00000650122.1 | 1.0534 | 0.0052 | 1.3443 | 0.0044 | Up |
| LINC02367 | ENST00000652814.1 | 1.1448 | 0.0016 | 1.6653 | 0.0289 | Up |
| C2orf27A | ENST00000653458.1 | 1.3176 | 0 | 1.1325 | 0.0113 | Up |
| LINC02368 | ENST00000654850.1 | 1.1001 | 0.0061 | 1.3835 | 0.0047 | Up |
| AL158214.2 | ENST00000657140.1 | 1.068 | 7.00E-04 | 1.0592 | 0.0015 | Up |
| CCDC18-AS1 | ENST00000657560.1 | 1.0667 | 0.0102 | 1.2608 | 0.0219 | Up |
| MIR4500HG | ENST00000658270.1 | 1.1592 | 0.0048 | 1.1008 | 0.0131 | Up |
| LINC01524 | ENST00000660314.1 | 1.1734 | 0.021 | 1.1004 | 0.0435 | Up |
| AC009236.1 | ENST00000663266.1 | 1.1837 | 0.0393 | 1.0847 | 0.0094 | Up |
| AFDN-DT | ENST00000663809.1 | 1.161 | 0.0013 | 1.207 | 0.0045 | Up |
| AFDN-DT | ENST00000669546.2 | 1.0708 | 0.0158 | 1.6487 | 0.0257 | Up |
| AL672310.1 | ENST00000670727.1 | 1.0718 | 0.0026 | 1.1176 | 0.023 | Up |
| AL359643.3 | ENST00000671039.1 | 1.0911 | 0.0038 | 1.5558 | 0.0014 | Up |
| FAM138C | ENST00000305248.5 | 0.9359 | 4.00E-04 | 0.9811 | 0.0403 | Down |
| WDFY3-AS2 | ENST00000318186.8 | 0.9447 | 0.0028 | 0.8096 | 0.05 | Down |
| LINC01554 | ENST00000357880.8 | 0.8342 | 7.00E-04 | 0.592 | 0.0079 | Down |
| AC007389.1 | ENST00000377977.3 | 0.936 | 0.0397 | 0.9122 | 0.0163 | Down |
| AC116351.1 | ENST00000399869.1 | 0.8758 | 0 | 0.7001 | 0.0014 | Down |
| FAM27C | ENST00000399894.6 | 0.894 | 0.036 | 0.4405 | 0.0039 | Down |
| LINC00598 | ENST00000400432.4 | 0.8097 | 0.0015 | 0.5653 | 3.00E-04 | Down |
| AL008626.1 | ENST00000412357.1 | 0.9596 | 0.0121 | 0.9657 | 0.0277 | Down |
| HCG14 | ENST00000412400.1 | 0.9529 | 0.0132 | 0.9638 | 0.0131 | Down |
| AC096531.2 | ENST00000413519.1 | 0.8999 | 0.004 | 0.7155 | 2.00E-04 | Down |
| LINC00421 | ENST00000413833.1 | 0.8921 | 0.0018 | 0.973 | 0.0298 | Down |
| AL513165.1 | ENST00000413915.1 | 0.9582 | 0.0397 | 0.9227 | 0.0182 | Down |
| MATN1-AS1 | ENST00000414532.6 | 0.9833 | 0.0029 | 0.8203 | 0.0151 | Down |
| MIR503HG | ENST00000414769.2 | 0.9239 | 1.00E-04 | 0.7547 | 2.00E-04 | Down |
| AL353597.1 | ENST00000414875.2 | 0.964 | 0 | 0.7878 | 0.0314 | Down |
| LINC01620 | ENST00000415299.2 | 0.8774 | 0 | 0.6827 | 0.0106 | Down |
| LINC02593 | ENST00000417705.1 | 0.9358 | 0.0134 | 0.8189 | 0.01 | Down |
| LINC01507 | ENST00000417801.1 | 0.8015 | 0.0019 | 0.4784 | 0.0042 | Down |
| LINC01871 | ENST00000417930.1 | 0.9072 | 0.0249 | 0.788 | 0.012 | Down |
| AL078590.2 | ENST00000418837.6 | 0.8089 | 0.0068 | 0.6518 | 0.009 | Down |
| AL078581.2 | ENST00000419134.1 | 0.9732 | 0.0188 | 0.9382 | 0.0391 | Down |
| LINC00261 | ENST00000420070.1 | 0.978 | 0.0365 | 0.8973 | 0.0075 | Down |
| TRAF3IP2-AS1 | ENST00000420651.2 | 0.9746 | 2.00E-04 | 0.9471 | 7.00E-04 | Down |
| LINC00271 | ENST00000421378.3 | 0.9457 | 0.0275 | 0.9305 | 4.00E-04 | Down |
| AC018467.1 | ENST00000421581.1 | 0.9013 | 0.0392 | 0.842 | 5.00E-04 | Down |
| TUBA3FP | ENST00000422086.5 | 0.8871 | 0.0021 | 0.7622 | 0.0344 | Down |
| LINC01777 | ENST00000423197.2 | 0.9723 | 0.0091 | 0.9503 | 0.0077 | Down |
| LINC02624 | ENST00000424701.1 | 0.7406 | 0.0052 | 0.3562 | 0 | Down |
| PRR7-AS1 | ENST00000425316.3 | 0.9795 | 8.00E-04 | 0.9657 | 0.0241 | Down |
| AC013270.1 | ENST00000425578.1 | 0.9699 | 4.00E-04 | 0.9753 | 1.00E-04 | Down |
| AL450384.2 | ENST00000425669.1 | 0.9232 | 0.0086 | 0.7346 | 0.0014 | Down |
| AL162414.1 | ENST00000426204.1 | 0.9355 | 0.0061 | 0.5812 | 0.0044 | Down |
| LINC00407 | ENST00000426509.1 | 0.9286 | 0.0014 | 0.9532 | 5.00E-04 | Down |
| ZEB2-AS1 | ENST00000427278.8 | 0.9595 | 3.00E-04 | 0.6822 | 0.0049 | Down |
| RRN3P2 | ENST00000427965.6 | 0.9507 | 1.00E-04 | 0.914 | 0.0024 | Down |
| LINC00310 | ENST00000428914.2 | 0.9612 | 0.043 | 0.6276 | 2.00E-04 | Down |
| LINC01238 | ENST00000429456.1 | 0.9516 | 0 | 0.9456 | 0.0342 | Down |
| LINC02640 | ENST00000429634.1 | 0.8667 | 5.00E-04 | 0.6897 | 0.0438 | Down |
| AF165147.1 | ENST00000430247.1 | 0.7865 | 4.00E-04 | 0.5415 | 0.0098 | Down |
| DSCR10 | ENST00000432141.1 | 0.974 | 0.0129 | 0.8776 | 0.004 | Down |
| LINC01506 | ENST00000432148.5 | 0.9293 | 0.017 | 0.9479 | 6.00E-04 | Down |
| AC099568.1 | ENST00000432395.1 | 0.9418 | 0.0487 | 0.7904 | 0.0305 | Down |
| NCKAP5-AS2 | ENST00000432414.5 | 0.7997 | 0 | 0.6774 | 0.0063 | Down |
| AC018467.1 | ENST00000432612.1 | 0.8124 | 0 | 0.7249 | 0.0024 | Down |
| HAR1A | ENST00000433161.1 | 0.9783 | 0.04 | 0.4669 | 0.0011 | Down |
| AF165147.1 | ENST00000433310.6 | 0.7221 | 1.00E-04 | 0.7803 | 0.0133 | Down |
| GNG12-AS1 | ENST00000434072.1 | 0.8822 | 0.0079 | 0.7253 | 0.0026 | Down |
| LINC00163 | ENST00000434081.1 | 0.9721 | 0.0051 | 0.9745 | 0.0114 | Down |
| AL162231.2 | ENST00000434627.1 | 0.8715 | 0.0316 | 0.8985 | 0.028 | Down |
| ZBTB46-AS1 | ENST00000435912.1 | 0.9633 | 0.0123 | 0.6209 | 0.0021 | Down |
| LINC01818 | ENST00000437118.1 | 0.8789 | 0.0109 | 0.6786 | 0.0037 | Down |
| AC008269.1 | ENST00000438070.2 | 0.8513 | 0.0033 | 0.6286 | 0.0178 | Down |
| AL691459.1 | ENST00000438589.1 | 0.8701 | 0.0079 | 0.909 | 0.0023 | Down |
| SLC39A12-AS1 | ENST00000439319.5 | 0.8489 | 0.0047 | 0.8477 | 0.0229 | Down |
| FAM27C | ENST00000439760.1 | 0.864 | 4.00E-04 | 0.5531 | 0.0067 | Down |
| LINC02519 | ENST00000440168.1 | 0.9764 | 0.0226 | 0.9259 | 0.0121 | Down |
| AC024084.1 | ENST00000440744.2 | 0.8951 | 0.0024 | 0.8702 | 0.005 | Down |
| AC018467.1 | ENST00000440785.1 | 0.8918 | 0.0014 | 0.7092 | 8.00E-04 | Down |
| Z82196.2 | ENST00000440858.1 | 0.9074 | 3.00E-04 | 0.9594 | 5.00E-04 | Down |
| AL590666.3 | ENST00000441272.2 | 0.94 | 2.00E-04 | 0.9669 | 9.00E-04 | Down |
| MIR503HG | ENST00000441492.1 | 0.8569 | 0.0195 | 0.7582 | 1.00E-04 | Down |
| LINC01664 | ENST00000441544.2 | 0.946 | 0.034 | 0.876 | 0.0022 | Down |
| AL133387.1 | ENST00000442188.1 | 0.8861 | 0.0054 | 0.9613 | 0.015 | Down |
| AL359764.2 | ENST00000444330.1 | 0.9886 | 0.0161 | 0.9309 | 7.00E-04 | Down |
| AL353804.1 | ENST00000444482.1 | 0.945 | 0.0055 | 0.761 | 0.0176 | Down |
| MIR503HG | ENST00000445415.1 | 0.7898 | 0.0033 | 0.5373 | 1.00E-04 | Down |
| AP003774.2 | ENST00000447028.1 | 0.9773 | 0.0119 | 0.9536 | 0.001 | Down |
| AL133319.1 | ENST00000447848.1 | 0.7761 | 0.0085 | 0.8435 | 4.00E-04 | Down |
| AC113608.2 | ENST00000448379.1 | 0.9709 | 0.0114 | 0.9329 | 0.0262 | Down |
| LINC01659 | ENST00000449711.1 | 0.9534 | 9.00E-04 | 0.9022 | 0.0035 | Down |
| LINC00322 | ENST00000450205.1 | 0.9769 | 0.0245 | 0.9543 | 0.0037 | Down |
| LINC01767 | ENST00000451914.2 | 0.8781 | 0.001 | 0.8664 | 0.0033 | Down |
| HNF4A-AS1 | ENST00000452481.1 | 0.935 | 0.0206 | 0.908 | 0.0023 | Down |
| AC092809.2 | ENST00000453760.5 | 0.8946 | 1.00E-04 | 0.758 | 0.0478 | Down |
| AP001471.1 | ENST00000454245.1 | 0.9304 | 0.009 | 0.862 | 0.0115 | Down |
| AL353803.2 | ENST00000454408.1 | 0.9886 | 0.044 | 0.9103 | 0.0316 | Down |
| AL161785.1 | ENST00000455981.1 | 0.8587 | 9.00E-04 | 0.7092 | 0.0072 | Down |
| LBX1-AS1 | ENST00000456391.1 | 0.9322 | 0.0012 | 0.9574 | 0.0102 | Down |
| LINC00885 | ENST00000457079.2 | 0.9808 | 0.038 | 0.788 | 0.0016 | Down |
| GAS6-AS1 | ENST00000458001.2 | 0.883 | 0.0035 | 0.9742 | 0.005 | Down |
| AC007731.2 | ENST00000458154.1 | 0.9834 | 0.0074 | 0.9482 | 0.0378 | Down |
| AC009951.6 | ENST00000475115.3 | 0.8581 | 4.00E-04 | 0.9646 | 0.0235 | Down |
| LINC02027 | ENST00000482617.1 | 0.5957 | 2.00E-04 | 0.579 | 0.0492 | Down |
| AC107029.1 | ENST00000484675.1 | 0.769 | 0 | 0.9101 | 0.0209 | Down |
| ADAMTS9-AS2 | ENST00000485174.5 | 0.9177 | 0.0071 | 0.7502 | 0.0412 | Down |
| MIR29B2CHG | ENST00000487977.2 | 0.9302 | 0.0272 | 0.963 | 0.0011 | Down |
| LINC01804 | ENST00000492352.5 | 0.911 | 0.0032 | 0.9571 | 0.0342 | Down |
| AC093010.2 | ENST00000493033.1 | 0.9733 | 0.0024 | 0.5988 | 0.0156 | Down |
| SUCLG2-AS1 | ENST00000496640.2 | 0.9268 | 0.0023 | 0.8402 | 0.0068 | Down |
| LINC01550 | ENST00000499006.7 | 0.9597 | 0.0319 | 0.4178 | 0.0047 | Down |
| SOCS2-AS1 | ENST00000499137.6 | 0.881 | 1.00E-04 | 0.9342 | 5.00E-04 | Down |
| A2M-AS1 | ENST00000499762.2 | 0.8961 | 9.00E-04 | 0.8506 | 0.0022 | Down |
| LINC00920 | ENST00000499966.1 | 0.8416 | 0.0037 | 0.5918 | 0.0056 | Down |
| LIFR-AS1 | ENST00000500733.6 | 0.7635 | 0.0078 | 0.8831 | 0.0141 | Down |
| AF233439.1 | ENST00000500823.3 | 0.9522 | 0.0213 | 0.9634 | 0.0018 | Down |
| LINC00861 | ENST00000500989.2 | 0.9174 | 0.0275 | 0.9403 | 0.0058 | Down |
| AC021491.1 | ENST00000502300.1 | 0.9555 | 2.00E-04 | 0.8092 | 0.0417 | Down |
| HAND2-AS1 | ENST00000502896.5 | 0.6882 | 1.00E-04 | 0.4813 | 1.00E-04 | Down |
| HAND2-AS1 | ENST00000502941.5 | 0.7296 | 5.00E-04 | 0.3829 | 1.00E-04 | Down |
| HHIP-AS1 | ENST00000503066.1 | 0.8101 | 0 | 0.7176 | 3.00E-04 | Down |
| LINC02268 | ENST00000503140.1 | 0.775 | 7.00E-04 | 0.8646 | 0.0033 | Down |
| LINC01612 | ENST00000504509.2 | 0.8883 | 0 | 0.4845 | 1.00E-04 | Down |
| AC093722.1 | ENST00000504882.1 | 0.9569 | 0.007 | 0.9899 | 0.0483 | Down |
| AC091173.1 | ENST00000505627.2 | 0.9614 | 0.0042 | 0.95 | 0.0016 | Down |
| FZD10-AS1 | ENST00000505807.6 | 0.9003 | 0.0226 | 0.9383 | 0.0202 | Down |
| AC098587.1 | ENST00000508241.1 | 0.9221 | 0 | 0.9661 | 0.0013 | Down |
| HHIP-AS1 | ENST00000508269.1 | 0.8663 | 5.00E-04 | 0.5053 | 1.00E-04 | Down |
| LINC01197 | ENST00000508732.6 | 0.871 | 1.00E-04 | 0.8735 | 0.0095 | Down |
| AC107396.1 | ENST00000509641.2 | 0.7608 | 2.00E-04 | 0.3764 | 0 | Down |
| CCDC26 | ENST00000509893.2 | 0.9008 | 0.0125 | 0.6723 | 0.0053 | Down |
| AC107223.1 | ENST00000510536.5 | 0.9304 | 0.0441 | 0.6813 | 7.00E-04 | Down |
| LINC02753 | ENST00000511013.2 | 0.8977 | 0.0196 | 0.8547 | 9.00E-04 | Down |
| HHIP-AS1 | ENST00000512359.1 | 0.5975 | 0 | 0.7792 | 0.0146 | Down |
| LINC02150 | ENST00000513157.1 | 0.9504 | 0.0173 | 0.7555 | 0.0419 | Down |
| AC112206.2 | ENST00000513234.5 | 0.9171 | 0 | 0.5792 | 0.0126 | Down |
| LINC00605 | ENST00000514902.2 | 0.9393 | 0.0036 | 0.7953 | 0.0067 | Down |
| RN7SKP283 | ENST00000517197.1 | 0.9513 | 0.0087 | 0.8698 | 0 | Down |
| SNORA50B | ENST00000517198.2 | 0.9684 | 0.0115 | 0.9259 | 0.0324 | Down |
| AL138963.2 | ENST00000517242.1 | 0.923 | 0.0022 | 0.9416 | 0.0482 | Down |
| CARMN | ENST00000524265.5 | 0.9089 | 0.0079 | 0.7711 | 0.0156 | Down |
| SENCR | ENST00000526269.2 | 0.9834 | 0.027 | 0.9666 | 0.0094 | Down |
| AL354813.1 | ENST00000526566.2 | 0.8586 | 0.0237 | 0.8442 | 0.0212 | Down |
| LINC02753 | ENST00000528316.5 | 0.8995 | 0.0251 | 0.8383 | 0.0031 | Down |
| AP003037.1 | ENST00000537594.1 | 0.7665 | 0 | 0.5787 | 0.0031 | Down |
| AC148477.2 | ENST00000537762.2 | 0.9507 | 2.00E-04 | 0.682 | 0.0111 | Down |
| LINC00612 | ENST00000538094.1 | 0.8989 | 0.0055 | 0.7638 | 0.028 | Down |
| LINC02361 | ENST00000538731.2 | 0.9383 | 0.0153 | 0.8884 | 0.0367 | Down |
| SOX9-AS1 | ENST00000540802.1 | 0.8923 | 0.0027 | 0.357 | 0 | Down |
| IFNG-AS1 | ENST00000541715.5 | 0.7886 | 0.0041 | 0.5905 | 0.0231 | Down |
| ROCR | ENST00000543512.1 | 0.8582 | 0.0021 | 0.3475 | 0 | Down |
| SOCS2-AS1 | ENST00000547845.5 | 0.8735 | 0 | 0.9282 | 0.0033 | Down |
| LINC02388 | ENST00000550678.1 | 0.8543 | 0 | 0.4698 | 0.0012 | Down |
| AC007298.2 | ENST00000551849.1 | 0.9136 | 6.00E-04 | 0.7066 | 0.0029 | Down |
| AL049836.2 | ENST00000553318.1 | 0.9068 | 0.0112 | 0.9202 | 0.0171 | Down |
| DIO3OS | ENST00000553575.1 | 0.8016 | 0.0113 | 0.5586 | 0.0104 | Down |
| LINC01197 | ENST00000555332.6 | 0.8614 | 1.00E-04 | 0.9466 | 0.0015 | Down |
| PSMA3-AS1 | ENST00000555707.5 | 0.9618 | 0.0061 | 0.9125 | 0.0055 | Down |
| LINC01197 | ENST00000556899.1 | 0.8644 | 0 | 0.7026 | 0.0188 | Down |
| AC012409.1 | ENST00000558449.1 | 0.888 | 0.0149 | 0.6035 | 0.0046 | Down |
| AC023024.1 | ENST00000558838.1 | 0.9571 | 0.0367 | 0.9002 | 2.00E-04 | Down |
| AC092755.2 | ENST00000560199.1 | 0.9589 | 0.0028 | 0.6797 | 0.0033 | Down |
| TAT-AS1 | ENST00000561529.1 | 0.8517 | 0.0013 | 0.8611 | 0.0253 | Down |
| PWRN1 | ENST00000562501.1 | 0.768 | 1.00E-04 | 0.7434 | 0.0141 | Down |
| AC009041.2 | ENST00000562570.1 | 0.9756 | 6.00E-04 | 0.8417 | 0.0118 | Down |
| AC010931.2 | ENST00000563727.1 | 0.9029 | 0.0041 | 0.9426 | 0.0464 | Down |
| AC099668.1 | ENST00000563780.1 | 0.9553 | 0.0488 | 0.9288 | 0.041 | Down |
| AC025259.3 | ENST00000564363.1 | 0.8481 | 0.005 | 0.901 | 0.0122 | Down |
| AC036108.3 | ENST00000564527.1 | 0.9587 | 0.0304 | 0.876 | 0.0184 | Down |
| AC009093.1 | ENST00000566070.1 | 0.9786 | 0.0049 | 0.8849 | 0.046 | Down |
| AC092375.2 | ENST00000567370.1 | 0.89 | 0.0119 | 0.6895 | 0.0036 | Down |
| AC115619.1 | ENST00000567376.2 | 0.9372 | 0.0059 | 0.8661 | 0.0073 | Down |
| AC026461.1 | ENST00000567563.1 | 0.8621 | 2.00E-04 | 0.4881 | 1.00E-04 | Down |
| AC104083.1 | ENST00000569449.1 | 0.8753 | 0.0055 | 0.8475 | 0.0133 | Down |
| LINC02192 | ENST00000570024.1 | 0.932 | 0.0065 | 0.9601 | 2.00E-04 | Down |
| AC093010.3 | ENST00000570269.2 | 0.9644 | 0.0056 | 0.907 | 0.0121 | Down |
| AC129507.1 | ENST00000570711.5 | 0.7421 | 1.00E-04 | 0.7687 | 2.00E-04 | Down |
| AC015909.3 | ENST00000572855.1 | 0.9795 | 7.00E-04 | 0.8207 | 0.0024 | Down |
| AL022341.2 | ENST00000573609.2 | 0.987 | 0.0221 | 0.7537 | 0.0197 | Down |
| AC087190.3 | ENST00000574616.2 | 0.9723 | 0.024 | 0.9299 | 0.0024 | Down |
| AC130343.1 | ENST00000576540.1 | 0.9666 | 0.009 | 0.9213 | 0.004 | Down |
| LINC02128 | ENST00000576842.1 | 0.8446 | 0.0467 | 0.7693 | 0.0018 | Down |
| AP005212.4 | ENST00000577614.2 | 0.8641 | 6.00E-04 | 0.8393 | 0.0211 | Down |
| PCAT18 | ENST00000579458.1 | 0.8522 | 0.0175 | 0.5046 | 7.00E-04 | Down |
| RARA-AS1 | ENST00000581080.1 | 0.8724 | 0.0026 | 0.8507 | 0.0114 | Down |
| AC024267.1 | ENST00000582320.2 | 0.9879 | 0.0348 | 0.8546 | 0.0029 | Down |
| DSG2-AS1 | ENST00000583706.5 | 0.9358 | 0.0049 | 0.8694 | 0.0365 | Down |
| AC093484.3 | ENST00000583934.1 | 0.9646 | 0.0022 | 0.9614 | 0.0015 | Down |
| LINC00907 | ENST00000585627.5 | 0.751 | 0.023 | 0.9194 | 0.0235 | Down |
| AC012615.2 | ENST00000586259.1 | 0.9761 | 1.00E-04 | 0.9416 | 1.00E-04 | Down |
| AC016590.1 | ENST00000586324.2 | 0.9847 | 0.0316 | 0.8998 | 0.0192 | Down |
| AC020907.2 | ENST00000586871.5 | 0.8465 | 6.00E-04 | 0.8471 | 0.0366 | Down |
| AC020931.1 | ENST00000589622.1 | 0.863 | 4.00E-04 | 0.88 | 0 | Down |
| AC025278.1 | ENST00000593558.1 | 0.8742 | 0.0032 | 0.94 | 0.0118 | Down |
| AC063977.6 | ENST00000600074.1 | 0.8709 | 0.0067 | 0.8386 | 0.0143 | Down |
| AC008555.4 | ENST00000603718.2 | 0.944 | 0.0135 | 0.8763 | 0.0022 | Down |
| AC025279.1 | ENST00000604430.1 | 0.9523 | 0.0022 | 0.7323 | 0.0084 | Down |
| FGF14-AS2 | ENST00000606448.1 | 0.9043 | 9.00E-04 | 0.9316 | 1.00E-04 | Down |
| AC087623.3 | ENST00000606593.1 | 0.8379 | 0.0035 | 0.9103 | 0.0481 | Down |
| AC104794.5 | ENST00000607181.1 | 0.9388 | 0.0077 | 0.8398 | 0.0038 | Down |
| AC245041.2 | ENST00000610809.1 | 0.8732 | 1.00E-04 | 0.9518 | 0.0058 | Down |
| LINC01197 | ENST00000611265.5 | 0.8586 | 1.00E-04 | 0.9604 | 0.0048 | Down |
| AL513548.3 | ENST00000611708.1 | 0.928 | 0.0011 | 0.9651 | 4.00E-04 | Down |
| AC015922.3 | ENST00000612568.1 | 0.9526 | 1.00E-04 | 0.8318 | 0.0482 | Down |
| AC005696.4 | ENST00000614400.1 | 0.9769 | 0.0068 | 0.8659 | 0.0041 | Down |
| AL162497.1 | ENST00000615635.1 | 0.9755 | 4.00E-04 | 0.9359 | 0.0279 | Down |
| AL034397.3 | ENST00000618234.4 | 0.835 | 0.0034 | 0.7452 | 0.0243 | Down |
| AC012409.2 | ENST00000619812.1 | 0.8525 | 0.0088 | 0.8401 | 0 | Down |
| EPB41L4A-DT | ENST00000623705.1 | 0.7999 | 1.00E-04 | 0.8389 | 0.019 | Down |
| LINC00891 | ENST00000627173.1 | 0.9699 | 0.0204 | 0.7893 | 0.0017 | Down |
| CPS1-IT1 | ENST00000628368.1 | 0.9362 | 0.0229 | 0.6251 | 0.0079 | Down |
| AC093627.11 | ENST00000632057.1 | 0.9197 | 0.0096 | 0.8658 | 0.0155 | Down |
| AC026950.1 | ENST00000633018.1 | 0.8568 | 0.0012 | 0.3484 | 0.0023 | Down |
| AC243651.1 | ENST00000633336.1 | 0.9698 | 0.0046 | 0.7868 | 0.0371 | Down |
| AC011139.1 | ENST00000635791.1 | 0.8009 | 0.0091 | 0.6523 | 0.0026 | Down |
| LINC00598 | ENST00000636621.1 | 0.8095 | 6.00E-04 | 0.4741 | 0.015 | Down |
| AC020912.1 | ENST00000638312.1 | 0.9762 | 0.0037 | 0.8371 | 0.0044 | Down |
| AL158064.1 | ENST00000638633.1 | 0.9213 | 0.0035 | 0.6895 | 5.00E-04 | Down |
| Z84466.1 | ENST00000638776.2 | 0.7599 | 0 | 0.728 | 8.00E-04 | Down |
| AC005906.2 | ENST00000639005.1 | 0.9626 | 0.0284 | 0.972 | 0.0461 | Down |
| AL391117.1 | ENST00000640003.1 | 0.9324 | 0.0117 | 0.7247 | 0.0299 | Down |
| LINC01488 | ENST00000642898.1 | 0.9265 | 0.0019 | 0.4369 | 0.0012 | Down |
| HULC | ENST00000646431.1 | 0.9579 | 0.004 | 0.7751 | 0.0132 | Down |
| LINP1 | ENST00000649087.1 | 0.9438 | 0.0281 | 0.9507 | 0 | Down |
| LINC02838 | ENST00000649656.1 | 0.967 | 6.00E-04 | 0.9437 | 3.00E-04 | Down |
| BX640514.2 | ENST00000650706.1 | 0.9669 | 6.00E-04 | 0.9675 | 0.0404 | Down |
| PWRN1 | ENST00000650918.1 | 0.759 | 1.00E-04 | 0.4761 | 2.00E-04 | Down |
| AC011479.4 | ENST00000651064.1 | 0.8633 | 0.0034 | 0.695 | 0.0327 | Down |
| PWRN1 | ENST00000651215.1 | 0.8855 | 2.00E-04 | 0.6363 | 0.0036 | Down |
| AL157394.3 | ENST00000651408.1 | 0.9214 | 0.0025 | 0.9447 | 0.0461 | Down |
| PWRN1 | ENST00000651558.1 | 0.802 | 0 | 0.6954 | 0.0356 | Down |
| PWRN1 | ENST00000652139.1 | 0.9547 | 0.0013 | 0.8003 | 8.00E-04 | Down |
| AC016682.1 | ENST00000652679.1 | 0.7628 | 0.0053 | 0.4857 | 0.0012 | Down |
| LINC01625 | ENST00000654256.1 | 0.8781 | 0.0252 | 0.5466 | 1.00E-04 | Down |
| AL360178.1 | ENST00000655005.1 | 0.7205 | 0.0038 | 0.4701 | 0.0037 | Down |
| AC012368.1 | ENST00000655199.1 | 0.9303 | 0.0327 | 0.8745 | 0.0137 | Down |
| AC010883.2 | ENST00000658582.1 | 0.976 | 0.0165 | 0.8635 | 0.006 | Down |
| AC006369.1 | ENST00000659653.1 | 0.9063 | 0.0022 | 0.6884 | 0.0177 | Down |
| DUBR | ENST00000660597.1 | 0.9566 | 0.0012 | 0.9723 | 0.03 | Down |
| AC098969.2 | ENST00000661256.1 | 0.9559 | 0.0302 | 0.9161 | 0.0031 | Down |
| AC090241.2 | ENST00000661793.1 | 0.885 | 0.0011 | 0.9255 | 1.00E-04 | Down |
| AC010280.1 | ENST00000663729.1 | 0.6602 | 0 | 0.5167 | 0.002 | Down |
| AC022075.1 | ENST00000665123.1 | 0.8367 | 0.001 | 0.7256 | 0.0025 | Down |
| AC104451.2 | ENST00000665745.1 | 0.9234 | 5.00E-04 | 0.9519 | 0.0271 | Down |
| C11orf40 | ENST00000668205.1 | 0.8677 | 0.0025 | 0.9377 | 9.00E-04 | Down |
| LINC02440 | ENST00000668907.1 | 0.9643 | 0.0299 | 0.9511 | 2.00E-04 | Down |
| UXT-AS1 | ENST00000669654.1 | 0.9356 | 0.0155 | 0.9345 | 0.0017 | Down |

**Supplementary Table 3 The relative expressions of *DYNLL1-AS1* and *RP11-116D2.1* between 14 HCC and adjacent normal samples.**

| PatientID | Cancer | | Adajcent normal | |
| --- | --- | --- | --- | --- |
|  | DYNLL1-AS1 | RP11-116D2.1 | DYNLL1-AS1 | RP11-116D2.1 |
| H1 | 1.8281 | 1.2772 | 0.8489 | 0.7490 |
| H2 | 21.8030 | 1.1982 | 1.2986 | 9.7842 |
| H3 | 2.2674 | 1.1625 | 0.5533 | 3.1854 |
| H4 | 3.5594 | 0.9933 | 0.3750 | 4.7680 |
| H5 | 2.4413 | 1.1665 | 0.8236 | 0.7827 |
| H6 | 1.7839 | 0.5329 | 0.9057 | 1.6803 |
| H7 | 431.4222 | 1.2504 | 1.0093 | 94.8383 |
| H8 | 0.2934 | 1.1462 | 0.6609 | 0.4305 |
| H9 | 128.6941 | 0.9621 | 1.5289 | 0.4023 |
| H10 | 156.5215 | 0.9236 | 1.2733 | 4.5609 |
| H11 | 26.1581 | 1.4631 | 0.9190 | 45.1339 |
| H12 | 30.8903 | 0.8885 | 0.9723 | 53.9866 |
| H13 | 7.5811 | 2.8937 | 0.9034 | 28.6026 |
| H14 | 0.4606 | 3.3965 | 4.2041 | 0.4542 |
